# Synthesis of Nano-Zinc Oxide Loaded on Mesoporous Silica by Coordination Effect and Its Photocatalytic Degradation Property of Methyl Orange

**DOI:** 10.3390/nano8050317

**Published:** 2018-05-09

**Authors:** Zhichuan Shen, Hongjun Zhou, Huayao Chen, Hua Xu, Chunhua Feng, Xinhua Zhou

**Affiliations:** 1 School of Chemistry and Chemical Engineering, Zhongkai University of Agriculture and Engineering, Guangzhou 510220, China; szcraiser@163.com (Z.S.); huayao_011@163.com (H.C.); dlutxuhua@163.com (H.X.); 2 Guangzhou Key Lab for Efficient Use of Agricultural Chemicals, Guangzhou 510220, China; 3 School of Environment and Energy, South China University of Technology, Guangzhou 510220, China; chfeng@scut.edu.cn

**Keywords:** nano ZnO, mesoporous silica, salicylaldimine, coordination, photodegradation

## Abstract

Salicylaldimine-modified mesoporous silica (Sal-MCM-3 and Sal-MCM-9) was prepared through a co-condensation method with different amounts of added salicylaldimine. With the coordination from the salicylaldimine, zinc ions were impregnated on Sal-MCM-3 and Sal-MCM-9. Then, Zn-Sal-MCM-3 and Zn-Sal-MCM-9 were calcined to obtain nano-zinc oxide loaded on mesoporous silica (ZnO-MCM-3 and ZnO-MCM-9). The material structures were systematically studied by Fourier transform infrared spectroscopy (FTIR), N_2_ adsorption/desorption measurements, X-ray powder diffraction (XRD), zeta potential, scanning electron microscopy (SEM), transmission electron microscopy (TEM), X-ray photoelectron spectroscopy (XPS), ultraviolet diffused reflectance spectrum (UV-vis DRS), and thermogravimetry (TGA). Methyl orange (MO) was used to investigate the photocatalysis behavior of ZnO-MCM-3 and ZnO-MCM-9. The results confirmed that nano ZnO was loaded in the channels as well as the outside surface of mesoporous silica (MCM-41). The modification of salicylaldimine helped MCM-41 to load more nano ZnO on MCM-41. When the modification amount of salicylaldimine was one-ninth and one-third of the mass of the silicon source, respectively, the load of nano ZnO on ZnO-MCM-9 and ZnO-MCM-3 had atomic concentrations of 1.27 and 2.03, respectively. ZnO loaded on ZnO-MCM-9 had a wurtzite structure, while ZnO loaded on ZnO-MCM-3 was not in the same crystalline group. The blocking effect caused by nano ZnO in the channels reduced the orderliness of MCM-41. The photodegradation of MO can be divided in two processes, which are mainly controlled by the surface areas of ZnO-MCM and the loading amount of nano ZnO, respectively. The pseudo-first-order model was more suitable for the photodegradation process.

## 1. Introduction

Nano zinc oxide (nano ZnO), which is well known for its highly activity and abundance for numerous organocatalysis [1,2], has previously demonstrated its performance in many applications such as the antibacterial field and catalytic degradation field [3,4]. Especially in the field of photodegradation, nano ZnO has attracted extensive attention owing to its strong quantum confinement effects [5]. This nano semiconductor possesses a wide band gap energy as well as a large exciton binding energy of about 60 meV, which allows excitonic emissions even at room temperature [6,7,8,9]. Thus, it can harvest ultraviolet radiation and generate electron-hole pairs, which will react with oxygen, further leading to the formation of hydroxyl radicals, superoxide radical anions, and hydroperoxyl radicals [10]. However, unsupported nano ZnO has one major drawback as it usually aggregates during the photocatalytic process, which reduces its surface area and thereby reduces the generation of reactive oxygen for the degradation of reagents [11,12,13]. In order to enhance its dispersion, loading the nano ZnO on a high surface support such as mesoporous silica can solve this problem [14].

In the past decade, mesoporous silica had been considerably made as a structural basis for nanotechnological applications as it is porous, thermally stable, non-toxic and hydrothermally stable [15,16,17,18,19]. Furthermore, as it has uniform and tunable channels with a pore diameter ranging from 2~50 nm, it can also be used to control the size of nanoparticles and make them homodisperse during their formation [6]. A good dispersion can help nanoparticles such as ZnO and TiO_2_ reduce the light scattering so that they demonstrate better photocatalytic properties [20,21]. These semiconductor nanoparticles can be incorporated inside the mesoporous silica by using various synthesis methods [22]. Among them, the impregnation method is most commonly used to load nano ZnO on mesoporous silica, which can help the nano ZnO form better crystallinity and provide a more direct path for the transfer of electrons, thus increasing photodegradation efficiency [23,24]. However, the limited loading amount of pure mesoporous silica is due to the lack of suitable active adsorption sites, which is still a problem during impregnation [25]. To solve this problem, chemical modification should be introduced to the mesoporous silica.

The surface of mesoporous silica is full of silanol groups which makes it easy to graft by chemical groups. Functional group anchoring can be achieved by using a functionalized silane or a co-condensation of silane with the silica or a post grafting of silica [26]. The introduction of organic compounds offers mesoporous silica specific adsorption sites for combination with metal ions. Chouyyok [27] reported that the chelating diamines modification made the mesoporous silica enhance the copper ions adsorption capacity in the waste water application due to the π back-bonding between copper ions and Phen aromatic ring system. He [28] found that the GLYMO-IDA grafted mesoporous silica had a good capacity in the adsorption of nickel ions through its strong binding ability. Such functional modifications can build the chemical bond between mesoporous silica and metal ions with a vacant electron orbit to improve the loading amount of metal ions. Nevertheless, fewer studies have paid much attention to this method in the application of loading metal oxide onto mesoporous silica. Only two studies have reported that nano ZnO were loaded inside the mesoporous silica by functionalizing the surface of MCM-41 using ethylene diamine [29,30]. In this system, the loading amount of ZnO was more than that of using pure MCM-41. Hence, the method of using functionalized mesoporous silica to make more nano ZnO loaded on mesoporous silica requires more research.

In this work, salicylaldimine, a kind of Schiff base, was used to adsorb zinc ions as a precursor for ZnO because of their straightforward synthesis and stability as well as their strong coordination with metal ions. On the one hand, owing to the π-system consisting of a benzene ring, phenolic hydroxyl, and carbon-nitrogen double bond, the salicylaldimine can play a key role as a chelating ring [31]. On the other hand, because empty orbitals exist, zinc ions can be regarded as the electron acceptor. These two factors together form the coordination bond between the zinc ion and salicylaldimine. Vasile [32] used a similar Schiff base modified mesoporous silica to adsorb copper ions where the adsorption capacity was 0.92 mmol/g. Its molar ratio of Cu/N was 0.42, which was very close to the theoretical value of 0.5. Salicylaldimine can be synthesized through the reaction of 3-Aminopropyltriethoxysilane with salicylaldehyde [33,34]. Then, by using the synthetic method of co-condensation or post-synthesis, the ethyoxyl of salicylaldimine will react with surface silanol groups of mesoporous silica [35], so that a certain amount of salicylaldimine is grafted onto the mesoporous silica. According to our knowledge, using a Schiff base modified mesoporous silica to adsorb zinc ions as a precursor of nano ZnO has not yet been reported and this method is worth exploring.

Herein, nano ZnO was loaded onto the surface of MCM-41 by oxidizing the zinc ions caught by the salicylaldimine modified MCM-41. A series of characterization tests were undertaken to analyze the structure of the materials. The properties and the kinetic behavior of the system were also evaluated by photodegradating the methyl orange, a commonly used coloring agent pollutant [36].

## 2. Experimental

### 2.1. Chemicals

Cetyl trimethyl ammonium bromide (CTAB), tetraethyl orthosilicate (TEOS), 3-Aminopropyltriethyloxy silane (APTES), and salicylaldehyde were all obtained from Shanghai Aladdin Biochemical Reagents (Shanghai, China). Ethanol, dichloromethane, anhydrous magnesium sulfate, ammonia solution (wt = 25%), zinc nitrate, and methyl orange (MO) were obtained from Tianjin Damao Chemical Reagents (Tianjin, China). All chemicals were analytical grade and used as received without any further purification.

### 2.2. Synthesis

#### 2.2.1. Preparation of Salicylaldimine

**Figure 1:**
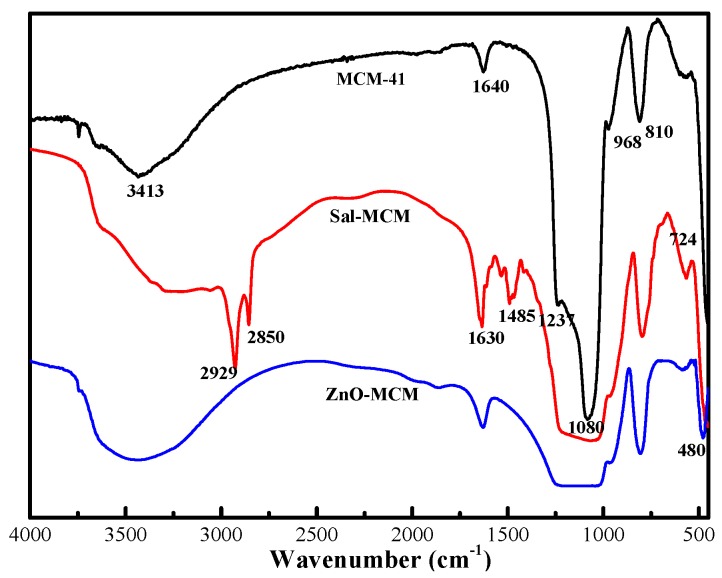
Fourier-transform infrared spectroscopy (FTIR) spectra of MCM-41, Sal, Sal-MCM, and ZnO-MCM.

According to the literature [34], 8.85 g (39.98 mmoL) of APTES, 4.88 g (39.96 mmoL) of salicylaldehyde, and 100 mL of ethanol were added to a flask and reacted at 95 °C for 3 h. After removing ethanol with rotary evaporation, the intermediate products were dissolved in 10 mL (156.01 mmoL) of dichloromethane. Then, the product was washed with deionized water three times. After being left to stand for 12 h, the organic layer was extracted, and 0.5 g (4.15 mmoL) anhydrous magnesium sulfate was used to remove the residual water. Finally, the final product was filtered to remove dichloromethane and magnesium sulfate to obtain salicylaldimine. According to FTIR (Figure 1), Peaks appearing at 780 cm^−1^ and 1630 cm^−1^ of Sal were attributed to the the vibration band of the benzene ring and the stretching band of C=N, which mean that the successful synthesis of salicylaldimine.

#### 2.2.2. Preparation of Salicylaldimine-Modified Mesoporous Silica (Sal-MCM)

As reported previously [37], the co-condensation synthesis method was used to prepare Sal-MCM. In a typical process, a total of 1.5 g (4.12 mmoL) of CTAB, 105 mL of ammonia solution (wt = 25%), and 150 mL of deionized water were added to the flask to be completely dissolved at 60 °C with 1 h of stirring. Then, 7.5 g (35.28 mmoL) of TEOS was added to the solution dropwise. After 1 h, 2.5 g of the as-synthesized salicylaldimine was added, and the reaction was stirred for another 6 h before being crystallized for 24 h at 30 °C, filtered, washed, and dried at 80 °C. At last, the template was removed with 100 mL ethanol at 110 °C for 72 h by the mean of reflux extraction to obtain Sal-MCM-41. Two kinds of samples were prepared and denoted as Sal-MCM-3 and Sal-MCM-9, which represented the modification amount of salicylaldimine as one-third and one-ninth of the mass of TEOS, respectively. Additionally, mesoporous silica without salicylaldimine modification was synthesized and noted as MCM-41.

#### 2.2.3. Preparation of Zinc Oxide Supported by Mesoporous Silica (ZnO-MCM)

**Scheme 1:**
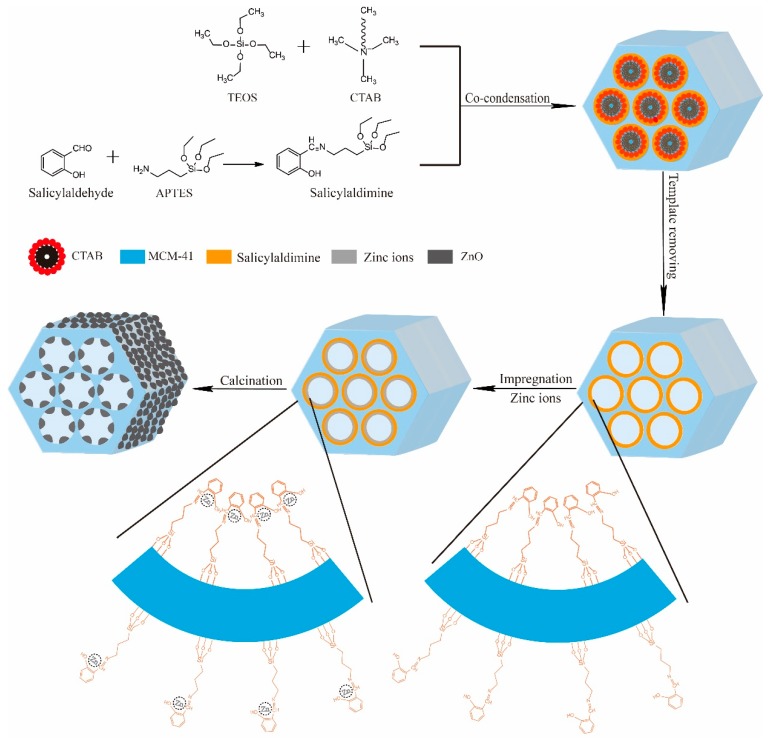
Schematic illustration of the synthetic process of ZnO-MCM.

Zinc oxide nanoparticles were loaded onto mesoporous silica through a wet impregnation method (Scheme 1 A total of 50 mL (100 mmoL) of zinc nitrate solution was added to 500 mg of Sal-MCM-41 or MCM-41 at 30 °C with stirring for 24 h. Then, Zn/Sal-MCM-41 and Zn/MCM-41 was obtained after being filtered, washed, and dried. Finally, following calcination at 550 °C for 6 h in air, ZnO-MCM was obtained. The samples were noted as ZnO-MCM-3, ZnO-MCM-9, and ZnO-MCM-0, where 3, 9, and 0 represent that the samples were prepared by Sal-MCM-3, Sal-MCM-9, and MCM-41, respectively.

### 2.3. Photocatalytic Degradation of MO and Reusablity Study

The photoefficiency of ZnO-MCM was evaluated through the degradation of an MO aqueous solution under UV-light irradiation. In a typical experiment, 50 mg of ZnO-MCM was dispersed in 100 mL of a 10 mg/L (0.003 mmoL) MO solution. Then, the suspension was exposed to a UV lamp (300 W, 365 nm wavelength) with stirring. To quantify the progress of the photodegradation reaction, 3 mL of sampling was taken out at 15 min intervals using a 1 mL pipette and centrifuged at 12,000 r/min; then, a 0.2 μm Millipore membrane filter was used to completely remove the residual ZnO-MCM nanoparticles to avoid its interference on subsequent MO absorbance analysis. A UV Lambda 365 ultraviolet-visible spectrometer from Perkin Elmer (Waltham, MA, USA) was applied to measure the amount of MO degraded by ZnO-MCM. A linear regression for the relationship between the solution concentration (*C*) and absorbance (*A*) of the MO standard solutions at different concentrations was performed at λ = 463 nm to obtain a standard curvilinear equation: *A* = 0.06746*C* + 0.04755; *R*^2^ = 0.99906. The efficiency of the photocatalytic activity was calculated using the following equation:

(1) $$DA=\frac{\left(C_{0}-C_{t}\right)}{C_{0}}\times 100\%$$

where *C*_0_ is the original mass concentration (mg/L) of the MO aqueous solution, and *C_t_* is the mass concentration (mg/L) of the MO aqueous solution at the relevant time interval. For contrast, the photocatalytic property of ZnO-MCM-0 and the self-degradation of MO were also examined.

The reusability of a selected ZnO-MCM sample was examined. For this purpose, the photocatalyst was filtered off after reaction, washed several times using distilled water, centrifuged (5000 r/min for 15 min), and subsequently dried at 80 °C in an oven. Then, it was reused as such for subsequent experiments (up to six cycles) under similar reaction conditions. For each cycle, the photocatalyst was reused for the photodegradation of a fresh MO solution. The same initial concentration of MO was used for each cycle (100 mL, 10 mg/L) and an irradiation time of 120 min was used.

### 2.4. Characterization

The samples were analyzed using a Bruker AXS D8 X-ray diffractometer (Bruker AXS GmbH, Karlsruhe, Germany) with Cu K*α* radiation (λ = 1.5418 Å) and a graphite monochromator at 25 °C, 40 kV, and 30 mA. The measurements were scanned at 2°/min (angular range 2θ = 0.5–5° and 5–90°) in a 0.02° step size. The morphology of the particles was analyzed by a Spectrum 100 Fourier infrared spectrometer (Perkin-Elmer, Waltham, MA, USA) using the KBr squash technique. Gold particles were sprayed onto the surface of the samples under the protection of N_2_, and the samples were characterized by a S4800 scanning electron microscope (Hitachi, Tokyo, Japan) to observe the surface topography. Transmission electron microscopy (TEM) observation was conducted on an FEI Tecnai G2 F20 transmission (Thermo Fisher Scientific, Waltham, MA, USA) election microscope. The Brunauer-Emmett-Teller (BET) surface area of the samples was determined by N_2_ adsorption isotherms at 77 K, operated on Quadrasorb SI adsorption equipment. The samples were degassed at 200 °C for 12 h in vacuum before the N_2_ adsorption experiment. The zeta potential and particle size of the samples were investigated with a Zetasizer Nano ZS (Bruker Corporation, Karlsruhe, Germany) in water at pH = 7 through ultrasonic dispersion. X-ray photo electron spectroscopy (XPS) were recorded on an ESCALAB 250Xi spectrometer (Thermo Fisher Scientific, Waltham, MA, USA, Al K*α*, *hν* = 1486.6 eV) under a vacuum of 2 × 10^−^^7^ Pa. Charging effects were corrected by adjusting the main C 1s peak to a position of 284.8 eV. A TGA 2 thermogravimetric analyzer (Mettler-Toledo, Columbus, OH, USA) was used to analyze the heat stability of the particles over the heating range of 40~800 °C and heating rate of 10 °C/min. The diffuse reflectance UV absorbance spectra of samples were recorded at 25 °C using a UV-3600 spectrophotometer (Shimadzu, Kyoto, Japan), all samples were measured with BaSO_4_ as the reference.

## 3. Results and Discussion

### 3.1. Characterization

In Figure 1, Fourier-transform infrared spectroscopy (FTIR) was adopted to compare the different compositions of MCM-41, Sal-MCM, and ZnO-MCM. For MCM-41, the bands located at 3413 cm^−1^ and 968 cm^−1^ were the stretching and bending vibrations of Si-OH, respectively [38]. The two bands that appeared at 1080 cm^−1^ and 810 cm^−1^ belonged to the characteristic peaks of Si-O-Si on the SiO_2_ framework. In comparison with MCM-41, two new bands appearing at 2929 cm^−1^ and 2850 cm^−1^ of Sal-MCM were attributed to the symmetric and non-symmetric C-H stretching vibration bands from the aminopropyl group. The vibration band of the benzene ring and the stretching band of C=N in salicylaldimine were located at 780 cm^−1^ and 1630 cm^−1^, respectively. It proved that the mesoporous silica was well modified by salicylaldimine. Compared with MCM-41, a red shift at 480 cm^−1^ of ZnO-MCM was observed, which indicated that ZnO was well incorporated in the channels of MCM-41 [39].

**Figure 2:**
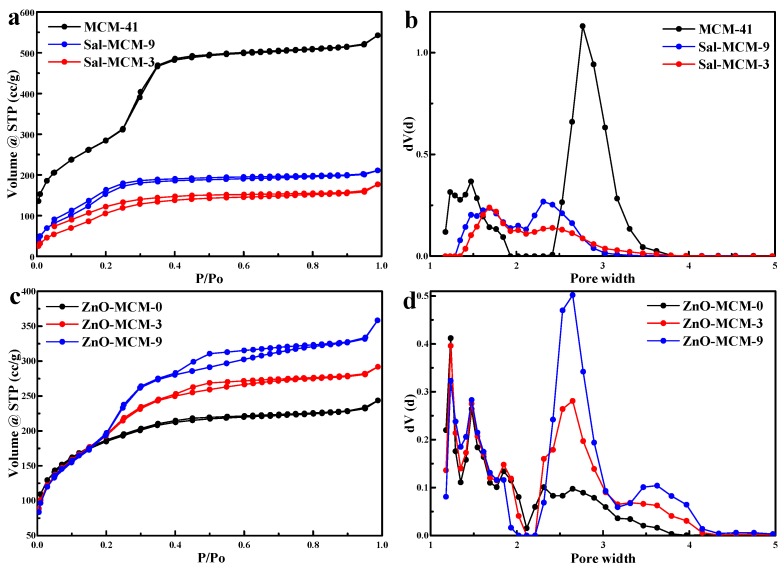
N_2_ adsorption/ desorption isotherms (**a**,**b**) and pore size distribution (**c**,**d**) of MCM-41, Sal-MCM, and ZnO-MCM.

As shown in Figure 2a, the N_2_ adsorption/desorption isotherms of MCM-41, Sal-MCM-9, and Sal-MCM-3 belonged to the Langmuir IV type adsorption. Hysteresis loops in these isotherms were not obvious, which meant that the samples possessed a small pore size [40], while the pore size distribution analyzed by the NLDFT method also confirmed the occurrence of micropores, as shown in Figure 2b. The adsorption and desorption branches of Sal-MCM could not be easily duplicated within *p*/*p*_o_ = 0.0 to *p*/*p*_o_ = 0.2 due to a certain extent of chemical adsorption by salicylaldimine during the testing process [41]. In addition, as shown in Table 1, the modification of salicylaldimine would significantly decrease the BET surface, pore diameter, and pore volume due to the blocking effect of salicylaldimine. Moreover, the BET surface of Sal-MCM-3 and Sal-MCM-9 were 453.385 m^2^/g and 634.437 m^2^/g, respectively, which meant that the salicylaldimine modification on Sal-MCM-3 was more than that of Sal-MCM-9. The modification of salicylaldimine not only decreased the pore size of the mesoporous silica, but also broadened the distribution.

As shown in Figure 2c, after the load of ZnO, the N_2_ adsorption/desorption isotherms of ZnO-MCM-0, ZnO-MCM-3 and ZnO-MCM-9 were still maintained as Langmuir IV types. Type H4 hysteresis loops were observed in the isotherms of ZnO-MCM-3 and ZnO-MCM-9 at *p*/*p*_o_ = 0.4, while the isotherm of ZnO-MCM-0 did not show any type of hysteresis loops. The appearance of the H4 hysteresis loop implied the instability of the adsorbed N_2_ due to the presence of ZnO in the pores of the mesoporous silica [40]. All the samples in Figure 2d showed three kinds of microporous distributions. Particularly, two mesopore systems, one with a relatively narrower and smaller pore size and another with a larger and broader pore size, were observed for ZnO-MCM-3 and ZnO-MCM-9. In contrast, ZnO-MCM-0 only exhibited a wider and smaller mesoporous distribution. It was supported that the introduction of ZnO would make the mesoporous silica form a hierarchical characteristic structure. As shown in Table 1, due to the blocking effect of ZnO, the BET surface, pore diameter, and pore volume decreased. Furthermore, when compared to MCM-41, the BET surface of ZnO-MCM-0, ZnO-MCM-3, and ZnO-MCM-9 decreased by about 561.72 m^2^/g, 447.834 m^2^/g, and 338.410 m^2^/g, respectively. It can be seen that the more salicylaldimine modifications the MCM-41 underwent, the more zinc ions would be adsorbed so that more nano ZnO would be supported on MCM-41. Additionally, it was obvious that both the changes of the BET surface of ZnO-MCM-3 and ZnO-MCM-9 were less than that of ZnO-MCM-0. This phenomenon proved that using the chemical groups modified on mesoporous silica to adsorb zinc ions would cause less damage to the surface areas of the vehicle than that of the simple physical adsorption of pure mesoporous silica before oxidizing zinc ions to ZnO.

**Figure 3:**
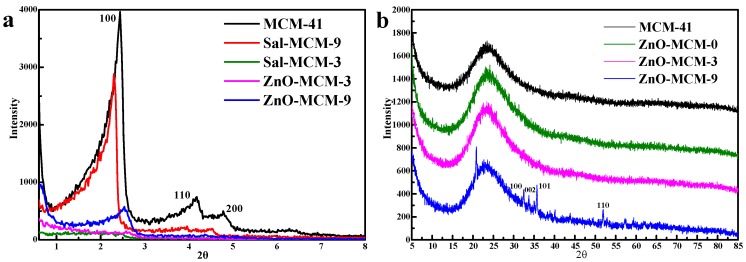
Low-X-ray diffraction patterns (XRD) (**a**) and Wide-XRD (**b**) patterns of MCM-41, Sal-MCM, and ZnO-MCM.

Figure 3 shows the low angle X-ray diffraction (LXRD) patterns of MCM-41, Sal-MCM-3, Sal-MCM-9, ZnO-MCM-3, and ZnO-MCM-9. Three characteristic peaks are shown in MCM-41, which could be ascribed to the (100), (110), and (200) crystal faces, indicating that the particles had a regular hexagonal pore structure [42]. The modification of salicylaldimine made the intensity of the low-angle XRD peaks decrease, especially in the (110) and (200) crystal face. Furthermore, when the amount of salicylaldimine increased, all of the crystal faces disappeared. All these phenomena identified that salicylaldimine was introduced to the system and decreased its degree of orderliness [43]. After the load of ZnO, peaks of the (100) crystal face of both ZnO-MCM-3 and ZnO-MCM-9 shifted to a higher 2θ, which meant the occurrence of ZnO in the channels. In comparison to Sal-MCM-9, the intensity of the (100) crystal face peak of ZnO-MCM-9 decreased, and the peaks of the (110) and (200) crystal faces faded away. However, the pattern of ZnO-MCM-3 still maintained the same shape as that of Sal-MCM-3. This result proved that the degree of orderliness of salicylaldimine-modified MCM-41 is crucial for ZnO formation inside the channels, and it further affects the orderliness of ZnO-MCM.

Figure 3 also shows the wide-angle X-ray diffraction patterns (WXRD) of MCM-41, ZnO-MCM-0, ZnO-MCM-3, and ZnO-MCM-9. Compared with MCM-41, no characteristic peaks of ZnO were observed in these wide-angle patterns of ZnO-MCM-0 and ZnO-MCM-3. Only diffuse peaks of the non-crystalline silica appeared, meaning that ZnO was finely loaded on the mesoporous silica and the cluster size of ZnO was too small to be detected by X-ray [44]. However, it also confirmed that ZnO was not present in the crystalline form [45], and the growth of ZnO was limited by the mesostructure of the vehicle. For ZnO-MCM-9, although the LXRD showed that the material was not in a good degree of orderliness, a series of peaks were still observed at the (100), (002), (101), and (110) crystal faces, which could be indexed to the wurtzite structure of ZnO [46]. Combined with the blocking effect confirmed by the N_2_ adsorption/desorption, it meant that the bad degree of orderliness of ZnO-MCM-9 was caused by ZnO. From the above information from the XRD patterns, it was supported that a good degree of orderliness of the vehicle as well as an appropriate amount of ZnO could help ZnO form a wurtzite structure on mesoporous silica.

As listed in Table 2, the zeta potential of Sal-MCM-3 shifted from −35.38 to 40.08 mV, owing to the positive ions from the nitric of salicylaldimine [47]. The result proved that the surface of MCM-41 was modified with salicylaldimine. Table 2 also shows that the zeta potential of ZnO-MCM-3 shifted from −35.38 to −23.08 mV. It proved that positively charged ZnO nanoparticles could partly neutralize the negative electric charges on the MCM-41 surface. However, Hassan [48] found that after the ZnO was supported on MCM-41, the material showed a positive zeta potential, which was about 9.3 mV. According to the detection mechanism of zeta potential, usually the electric potential detected by the machine was from the electrostatic field of the surface of the nanoparticles [49]. This confirmed that most of the ZnO nanoparticles were loaded in the channels of MCM-41 and only a small part of ZnO was loaded on the outside surface, which could also be proved from the change of pore diameter shown in Table 1.

**Figure 4:**
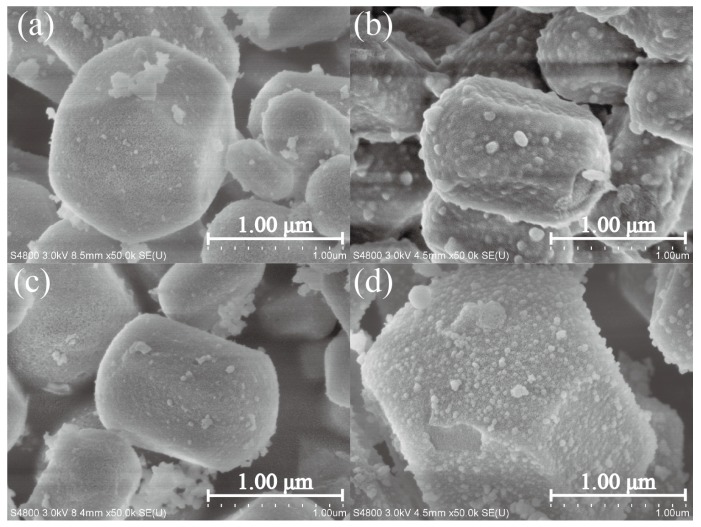
Scanning Electron Microscope (SEM) images of MCM-41 (**a**); Sal-MCM (**b**); ZnO-MCM-0 (**c**); and ZnO-MCM-9 (**d**).

**Figure 5:**
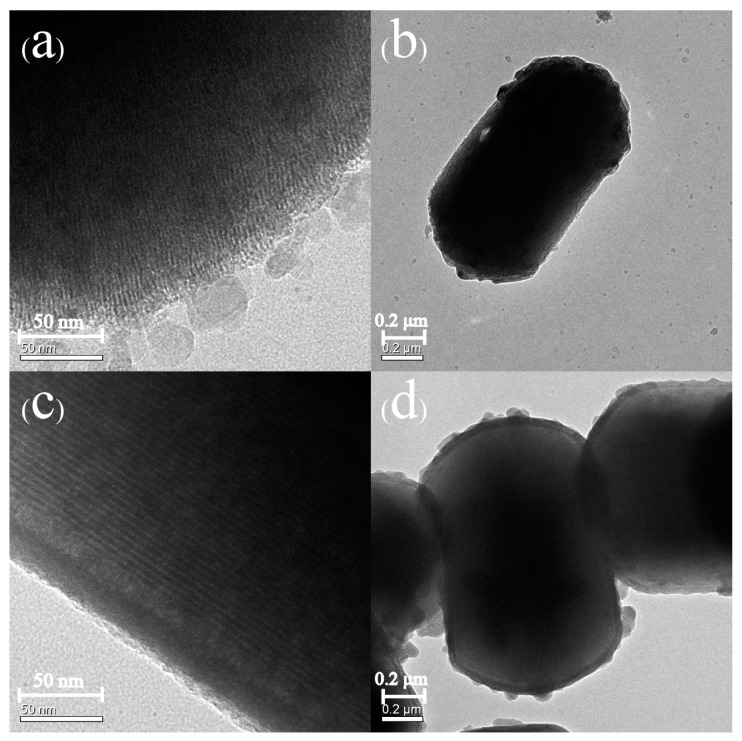

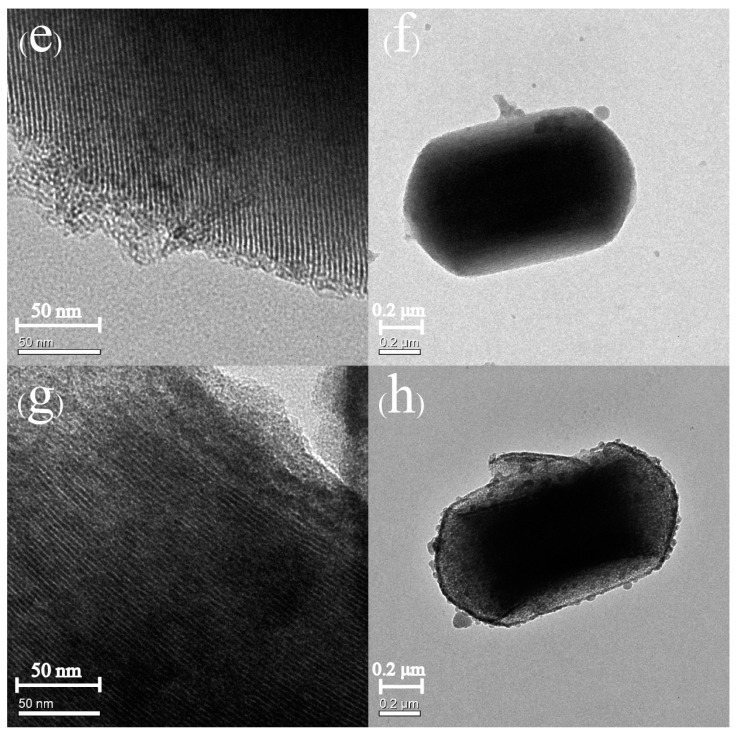
Transmission electron microscopy (TEM) images of MCM-41 (**a**,**b**), Sal-MCM (**c**,**d**), ZnO-MCM-0 (**e**,**f**), and ZnO-MCM-9 (**g**,**h**).

Figure 4 and Figure 5 depict the SEM and TEM images of MCM-41, Sal-MCM, ZnO-MCM-0, and ZnO-MCM-9. As shown, the regular hexagonal pore structure was well-maintained without agglomeration after the modification of salicylaldimine or the load of ZnO. Both Sal-MCM and ZnO-MCM showed a rough shell structure on the external surface with many circular particles due to the introduction of salicylaldimine and ZnO. The surface of ZnO-MCM-9 (Figure 4d) was rougher than that of ZnO-MCM-0 (Figure 4c), indicating that salicylaldimine could help the vehicles adsorb more zinc ions and, thus, more ZnO would form. Comparing the wurtzite form of nano ZnO showed by WXRD to the spherical form nanoparticles on the surface of ZnO-MCM-9, it was obvious that the wurtzite shaped ZnO were formed in the channels instead of the outside surface of MCM-41. This proved that the shape of ZnO nanoparticles could be controlled by the changeable channels of mesoporous silica, while those ZnO nanoparticles grown on the outside surface could not form in the same shape due to the lack of restrictions. Materials in Figure 5 all presented in the parallel lattice fringes structure [50] and corresponded to (100) crystal face, which showed the highest peak in Low-XRD.

**Figure 6:**
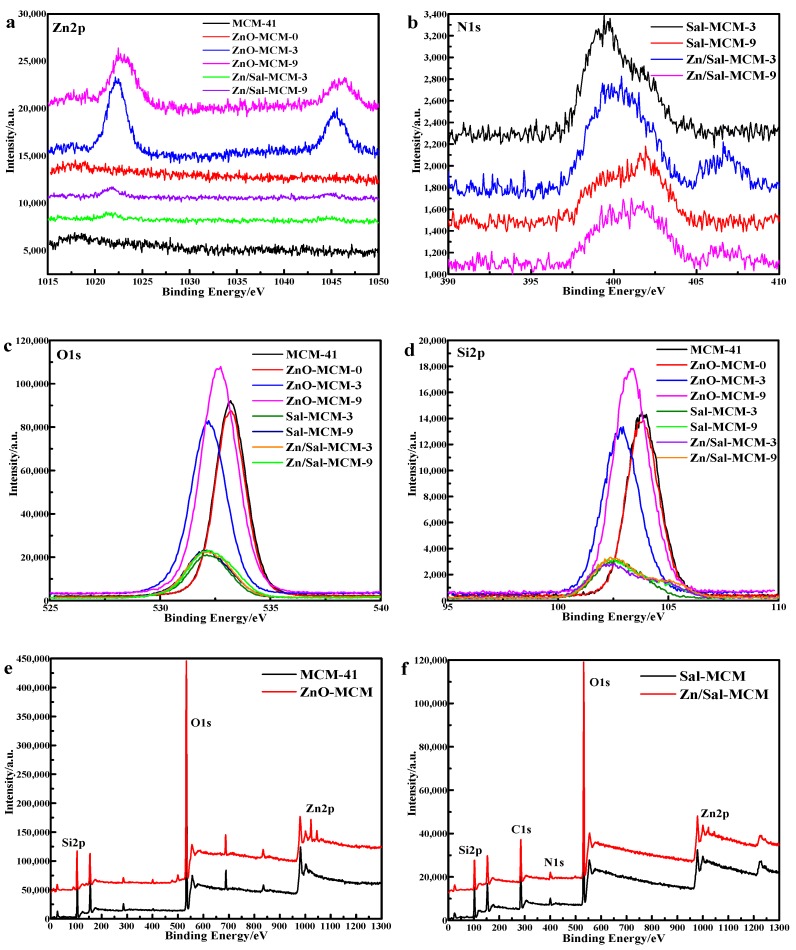
X-ray photo electron spectroscopy (XPS) of Zn 2p (**a**); N 1s (**b**); O 1s (**c**); Si 2p (**d**); survey (**e**,**f**) ofMCM-41, Sal-MCM, and ZnO-MCM.

**Figure 7:**
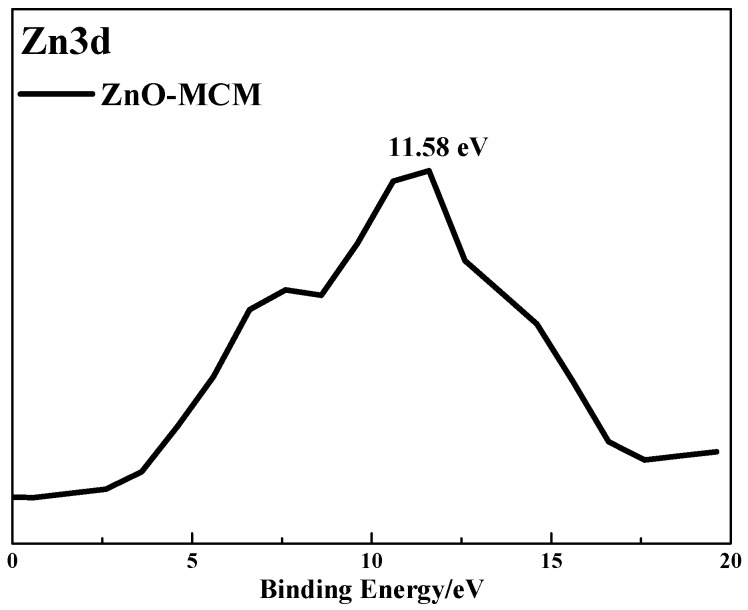
XPS of Zn 3d of ZnO-MCM.

As seen in Figure 6, the XPS analysis was carried out to investigate the nanoparticle composition. In Figure 6a, it was observed that the binding energy regions positioned at 1022 eV and 1045 eV were assigned to Zn 2p_3/2_ and Zn 2p_1/2_, respectively [51], from the spectrum lines of both of the ZnO-MCM-3 and ZnO-MCM-9. Zn/Sal-MCM-3 and Zn/Sal-MCM-9 showed two peaks of Zn 2p at 1021 eV and 1044 eV, respectively, which means that the zinc ions were coordinated with salicylaldimine. After the formation of ZnO, the peak at 1044 eV of Zn 2p_1/2_ was more obvious. Meanwhile, the peak at 1021 eV shifted positively to 1022 eV, due to the coordination bonds between zinc ions and salicylaldimine being replaced by the ionic bonds between the zinc atoms and oxygen atoms. On the one hand, the spectrum of ZnO-MCM-0 showed no special difference from that of MCM-41, while the spectrum lines of ZnO-MCM-3 and ZnO-MCM-9 were distinctly different from those of Sal-MCM-3 and Sal-MCM-9. On the other hand, for ZnO-MCM-0, its atomic concentration was 0.39, while ZnO-MCM-3 and ZnO-MCM-9 showed the atomic concentrations of 2.03 and 1.27, respectively (Table 3). These results confirmed that the more ZnO was loaded on MCM-41, the more obvious the peaks would be. It could be concluded that the modification of salicylaldimine does help to catch more zinc ions due to its strong coordination function. From Figure 7, we also found that the binding energy of the Zn 3d electron in ZnO-MCM is 11.58 eV, 1.93 eV higher than that of pure ZnO, which indicates the formation of Si-O-Zn bond. This proved that, after the calcination, the interaction between ZnO and the silica matrix is the Si-O-Zn bond [39].

Figure 6b exhibits the binding energy spectrum line of N 1s. A positive shift of Zn/Sal-MCM-3 was observed, which showed that the peak value of 399.44 eV moved to 400.14 eV. It was proposed that the electron transfer from nitrogen to zinc ions should be responsible for this result [52]. It proved that the coordination between the salicylaldimine and zinc ions was successfully formed.

In Figure 6c, all the binding energy peaks of O 1s were symmetrical, which was different from previous reports [50] that indicated that only one chemical state for the oxygen species existed. For MCM-41, a peak centered at 533.19 eV was definitely from the result of the oxygen atoms of MCM-41. Those energy peaks of O 1s, excluding that of ZnO-MCM-0, took a negative shift in varying degrees after the modification of salicylaldimine or ZnO. This means that the modification of chemical groups or metal oxides would disturb the electron distribution of the surface of the vehicles. After the ZnO formed on the MCM-41, the electron intensity of O 1s also increased, when compared to those of Sal-MCM-3 and Sal-MCM-9. It could be suggested that the zinc ions were transformed into ZnO because during this process, oxygen atoms would combine with zinc atoms, resulting in the increase of intensity of O 1s. In Figure 6d, the same change trend was also observed from the binding energy spectrum of Si 2p, which could also be attributed to the similar reason. The overall XPS spectrum of MCM-41, ZnO-MCM, Sal-MCM, and Zn/Sal-MCM are shown in Figure 6e,f. All of the spectrum results confirmed that ZnO was successfully loaded onto MCM-41.

**Figure 8:**
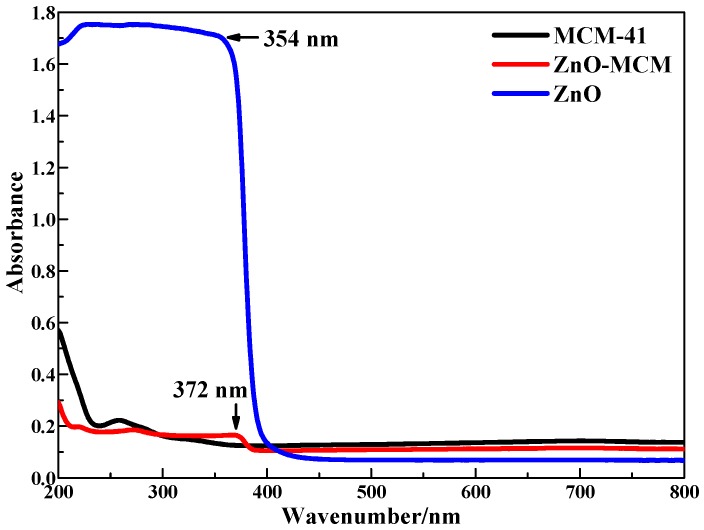
Diffuse reflectance UV-vis spectra of MCM-41, ZnO-MCM, and ZnO.

The UV-vis DRS of the nano-zinc oxide, mesoporous silica, and ZnO-MCM samples are shown in Figure 8. The adsorption band edge at 372 nm for ZnO-MCM suggests the presence of ZnO particles [6]. Compared with ZnO, a red shift of the adsorption band edge was observed, which can be ascribed to the well-known quantum size effect [53]. The wide absorption band from 250 to 372 nm observed in the ZnO-MCM sample might be due to the formation of one-dimensional array inside the mesoporous silica, which is expected for these types of crystal growth [39]. Based on the maximum absorption wavelength, the band gap of ZnO nanoparticles supported on mesoporous silica was calculated to be 3.33 eV according to the relation *E*_bg_ = 1240/λ_max_, while the band gap of pure nano-zinc oxide was 3.50 eV [54]. This means that the photocatalytic performance of ZnO was improved by being supported on mesoporous silica.

**Figure 9:**
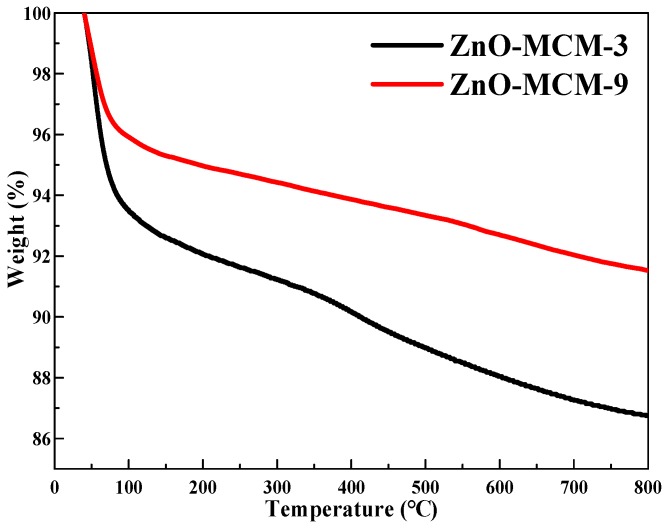
Thermogravimetry (TGA) curves of ZnO-MCM-3 and ZnO-MCM-9.

As shown in Figure 9, ZnO-MCM-3 and ZnO-MCM-9 were subjected to TGA to obtain the thermal stability information. For both ZnO-MCM-3 and ZnO-MCM-9, the loss in mass from 40 °C to 100 °C was due to the elimination of the crystallization water and residual physically adsorbed water on the surface of the channels [55]. The final residues of ZnO-MCM-3 and ZnO-MCM-9 were 86% and 91%, respectively, which showed that there were more nano zinc oxides supported on ZnO-MCM-3 than that of ZnO-MCM-9. It also proved that the more salicylaldimine was grafted on MCM-41, the more zinc ions would be coordinated and thus, more nano zinc oxides would form. According to the previous literature [56], the ZnO transformed into Zn_2_SiO_4_ began from 650 to 800 °C due to the reaction between ZnO and SiO_2_. However, in this work, the TG curves did not show an obvious sudden heat loss step at this temperature range and only a slow loss of weight during 100 to 800 °C was observed. It is supposed that the transformation of this material began at 100 °C and only a fraction of ZnO transform. Further research on this phenomenon will be undertaken in our future work.

### 3.2. Degradation of MO

**Figure 10:**
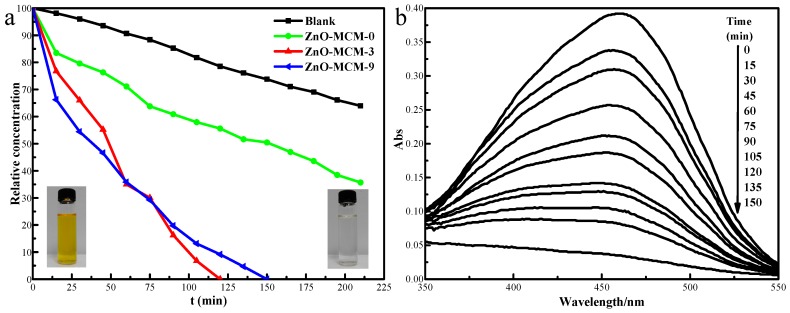
Photodegradation of MO under UV light using ZnO-MCM-0, ZnO-MCM-3 and ZnO-MCM-9 (**a**); and UV-Vis absorbance spectra of MO (**b**).

Figure 10 shows the photocatalytic activity of the as-prepared ZnO-MCM-0, ZnO-MCM-3, and ZnO-MCM-9 nanoparticles. The photodegradation of the MO was investigated in deionized water under ultraviolet irradiation. The MO solution without adding the as-prepared nanoparticles was chosen for comparison. The degradation process can be divided into two parts. During 45 min, the double effect of adsorption and degradation of ZnO-MCM-9 and ZnO-MCM-3 made the concentration of MO rapidly decline by 54% and 45%, respectively. From the 45 min to the end, the degradation rate caused by ZnO-MCM-3 was faster than that of ZnO-MCM-9. This could be indicated that in the first process of photocatalytic degradation, the surface area played a dominant role, while in the second process, the amount of ZnO loaded on MCM-41 dominated the degradation according to the zinc atomic weight of ZnO-MCM-3, which was more than that of ZnO-MCM-9 while the BET surface of ZnO-MCM-3 was smaller. Furthermore, the degree of orderliness as well as the crystallinity of the zinc oxide did not show any significant influence on the photocatalytic efficiency of ZnO-MCM. As a result, it took approximately 120 min to reduce the MO concentration using ZnO-MCM-3, while an additional 30 min was required with the use of ZnO-MCM-9. The pure MO degraded approximately 35% after 200 min, which implied that without ZnO-MCM, ultraviolet radiation only caused negligible degradation to MO.

The two photos inset in Figure 10a reflect the obvious change in the color of the MO solution with the addition of ZnO-MCM and the ultraviolet adsorption spectrum was used to confirm the degradation of MO. The color changed from orange to colorless in 150 min with the use of ZnO-MCM under UV light, and the characteristic peak at 463 nm for the absorption became weaker and disappeared in the final. This observation indicated that ZnO-MCM would accelerate the degradation of MO.

### 3.3. Degradation Kinetics Study

According to previous reports [39,50,57,58], a pseudo-second-order model (Equation (4)) [59] and pseudo-first-order model (Equation (3)) [60,61,62] evolved from the Langmuir−Hinshelwood mechanism (L-H model, Equation (2)) [63,64,65] can be applied to describe the photocatalytic degradation process of the organic dye reaction at the liquid-solid interface [66]. All the equations were displayed as follows:

(2) $$r=\frac{dC}{dt}=\frac{K_{r}K_{s}C}{1+K_{s}C_{o}}$$

At a low initial concentration of MO, Equation (2) can be transformed into Equation (3).

(3) $$\ln\left(\frac{C_{o}}{C_{t}}\right)=K_{app}t$$

(4) $$\frac{1}{C_{t}}=Kt+\frac{1}{C_{o}}$$

where *C_o_* and *C_t_* represent the concentration at the initial and treatment process time. *K_s_* and *K_r_* are the adsorption rate and reaction rate, respectively. *K_app_* and *K* are the apparent pseudo-first-order and second-order rate constants, respectively.

**Figure 11:**
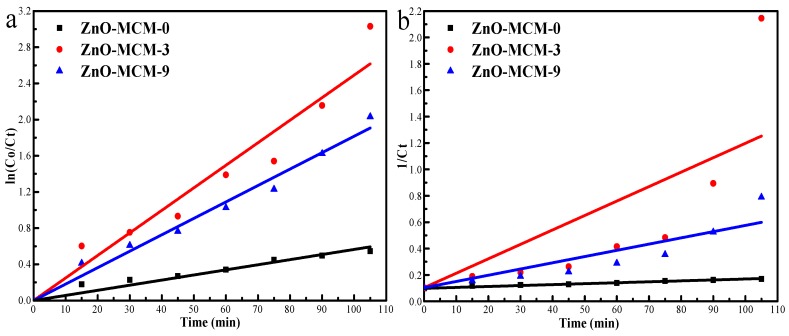
Photodegradation kinetics of MO using the pseudo-first-order model (**a**); and the pseudo-second-order model (**b**).

The data of the MO degradation were fitted to a pseudo-first-order model and a pseudo-second-order model (Figure 11). The R values and constants of the two models are listed in Table 4. It was obvious that the pseudo-first-order model was more suitable than the pseudo-second-order model for the MO degradation. For ZnO-MCM-0, ZnO-MCM-9, and ZnO-MCM-3, the *K_app_* values were 0.00565, 0.01816 and 0.02491, respectively, which indicated that the degradation rate was increasingly faster as the amount of ZnO increased.

### 3.4. Degradation Mechanism

**Scheme 2:**
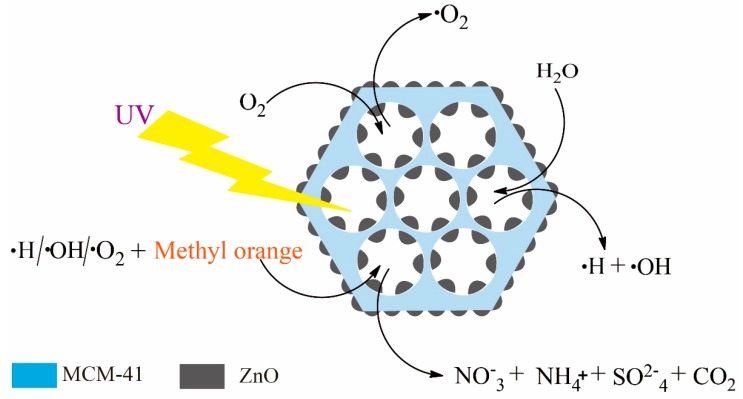
Schematic illustration of degradation mechanism.

A possible mechanism of the degradation behavior would help to better understand the photocatalysis result (Scheme 2). As previous reports have depicted [67], irradiation with a suitable UV-light source whose band gap energy is smaller than its photon energy would activate the photocatalyst [68]. When a photon is caught by zinc oxide, it facilitates an electron transfer from the valence band to the conduction band. At this moment, an electron-hole pair emerges. These electron-hole pairs migrate to the surface of ZnO and start to oxidize and reduce the MO molecules absorbed on the outside surface or in the channels of the ZnO-MCM [69]. The surface hydroxyl groups acquire the holes and strongly oxidize to form hydroxyl radicals, which are generated in this process. At the same time, the combined materials, which include the aqueous solution, the oxide and the oxide radicals, also produce hydroxyl radicals. Ultimately, hydroxyl radicals generated by these two processes allow the oxidation of the MO dye absorbed on the surface of ZnO, leading to the formation of ammonium, nitrate ion, sulfate, and CO_2_.

### 3.5. Reusability Performance

**Figure 12:**
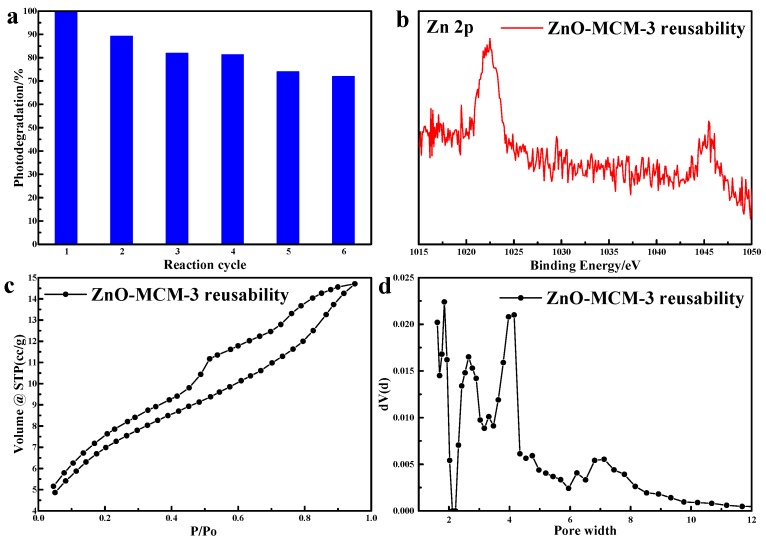
Reusability of ZnO-MCM-3 toward the photodegradation of MO (**a**), XPS of Zn 2p (**b**), N_2_ adsorption/desorption isotherms (**c**) and pore size distribution (**d**).

As shown in Figure 12, Table 5 and Table 6, after six cycles, ZnO-MCM-3 was still effective in the photodegradation of MO, remaining above 70%. The decrease in the photocatalytic activity might be due to the loss of zinc, the change of pore structure and the reduction of surface area. These change of parameters not only resulted in the change of the amount of active oxygen produced by ZnO-MCM-3, but also brought a decrease of the contact area between ZnO-MCM-3 and MO. All of the factors ultimately affected the photodegradation efficiency.

## 4. Conclusions

In this study, ZnO-MCM-3 and ZnO-MCM-9 with photodegradation properties were prepared by two steps. Zinc ions were introduced to MCM-41 by coordinating with salicylaldimine modified on mesoporous silica and then a high-temperature oxidation was undertaken to transform zinc ions into zinc oxide and remove the salicylaldimine. The characterization confirmed that during the wet impregnation, the coordination of salicylaldimine could help to adsorb more zinc ions so that more ZnO would load on mesoporous silica. The more salicylaldimine was grafted on MCM-41, the more zinc ions would be caught and more nano zinc oxide would form. The appearance of hierarchical tunnels proved that ZnO were loaded in the channels of MCM-41. Furthermore, a suitable grafted amount of salicylaldimine as well as a good degree of orderliness would help MCM-41 to make those nano ZnO loaded in the channels grow into wurtzite crystalline. However, due to the lack of restrictions, nano ZnO loaded on the outside surface of MCM-41 were formed in spherical instead of wurtzite crystalline. The addition of ZnO-MCM did enhance the degradation efficiency of MO under UV radiation. The degradation of MO was mainly controlled by two physical constants. The surface areas of the ZnO-MCM played a dominant role in the first process while the loading amount of nano ZnO showed a more important role during the second process. Meanwhile, though the blocking effect of nano ZnO in the channels would reduce the orderliness of the vehicle, it was seen that this effect did not have much influence on the degradation efficiency. The pseudo-first-order model could be used to explain the photodegradation process. In brief, with the help of the coordination effect of the modified mesoporous silica, more metal ions will be adsorbed, so that more nano metal oxide will load on the mesoporous silica after the oxidization process. This method will make the mesoporous materials play a more significant role in the loading of nano metal oxide.

## Figures and Tables

**Table 1:** The pore structural parameters of MCM-41, Sal-MCM, and ZnO-MCM.

Materials	Surface Area (m^2^/g)	Pore Diameter Dv (nm)	Pore Volume (m^3^/g)
MCM	1187.377	2.444	0.993
ZnO-MCM-0	625.657	0.887	0.429
Sal-MCM-3	453.385	1.934	0.388
ZnO-MCM-3	739.543	2.180	0.547
Sal-MCM-9	634.437	1.932	0.478
ZnO-MCM-9	848.967	2.176	0.682

**Table 2:** Zeta potential of MCM-41, Sal-MCM, and ZnO-MCM.

Materials	MCM-41	Sal-MCM-3	ZnO-MCM-3
Zeta Potential (mV)	−35.38	40.08	−23.08

**Table 3:** Binding energy and atomic of MCM-41, Sal-MCM, Zn/Sal-MCM, and ZnO-MCM.

Material	BE/eV				Atomic/%	
	Zn 2p	N 1s	Si 2p	O 1s	Zn 2p	Zn 2p3s
MCM	-	-	103.83	533.19	-	-
Sal-MCM-3	-	399.44	102.63	532.20	-	-
Sal-MCM-9	-	401.87	102.47	532.09	-	-
Zn/Sal-MCM-3	1021.26	400.14	102.40	532.17	-	0.334
Zn/Sal-MCM-9	1021.75	401.14	102.54	532.24	-	0.415
ZnO-MCM-0	1017.90	-	103.81	533.18	0.39	-
ZnO-MCM-3	1022.27	-	102.84	532.19	2.03	-
ZnO-MCM-9	1022.90	-	103.29	532.67	1.27	-

**Table 4:** Fitting results of the photodegradation of MO.

Kinetic Models	Fitting Equation	*R* ^2^
pseudo first-order	$\ln\frac{C_{o}}{C_{t}}=\left\{ \begin{array}{l} 0.00565t;\text{ZnO-MCM-0}\\ 0.02491t;\text{ZnO-MCM-3}\\ 0.01816t;\text{ZnO-MCM-9} \end{array}\right.$	0.98225
		0.97787
		0.99306
pseudo second-order	$\frac{1}{C_{t}}=\left\{ \begin{array}{l} 0.0007t+0.09882;\text{ZnO-MCM-0}\\ 0.01094t+0.10335;\text{ZnO-MCM-3}\\ 0.00472t+0.10335;\text{ZnO-MCM-9} \end{array}\right.$	0.99913
		0.74946
		0.93623

**Table 5:** The pore structural parameters of ZnO-MCM-3 after six photodegradation cycles of MO.

Materials	Surface Area (m^2^/g)	Pore Diameter Dv (nm)	Pore Volume (m^3^/g)
ZnO-MCM-3	739.543	2.180	0.547
Reusable ZnO-MCM-3	420.100	3.043	0.233

**Table 6:** Binding energy and atomic of ZnO-MCM-3 after six photodegradation cycles of MO.

Material	BE/eV				Atomic/%
	Zn 2p	N 1s	Si 2p	O 1s	Zn 2p
ZnO-MCM-3	1022.27	-	102.84	532.19	2.03
Reusable ZnO-MCM-3	1022.37	-	102.92	531.89	0.63
